# Rotavirus vaccines in Africa and Norovirus genetic diversity in children aged 0 to 5 years old: a systematic review and meta-analysis: Rotavirus vaccines in Africa and Norovirus genetic diversity

**DOI:** 10.1186/s12879-024-09434-6

**Published:** 2024-05-31

**Authors:** Dako Dakouo, Djénéba Ouermi, Abdoul Karim Ouattara, Abibou Simpore, Tégwendé Rebecca Compaore, Mah Alima Esther Traore, Zakaria Gamsore, Abdou Azaque Zoure, Lassina Traore, Théodora Mahoukèdè Zohoncon, Albert Théophane Yonli, P. Denise Ilboudo, Florencia Wendkuuni Djigma, Jacques Simpore

**Affiliations:** 1 Laboratoire de Biologie Moléculaire et Génétique (LABIOGENE), Université Joseph KI- ZERBO, Ouagadougou 03, 03 BP 7021 Burkina Faso; 2 Centre de Recherche Biomoléculaire Pietro Annigoni (CERBA), Ouagadougou 01, 01 BP 364 Burkina Faso; 3 Département de Biologie et Physiologie Animales, Université Joseph KI-ZERBO, Ouagadougou, Burkina Faso; 4 Agence Nationale pour la Sécurité Sanitaire de l’Environnement, de l’Alimentation, du Travail et des Produits de Santé (ANSSEAT), Ouagadougou, Burkina Faso; 5 Faculté de Médecine, Université Saint Thomas d’Aquin, Ouagadougou 01, 06 BP 10212 Burkina Faso; 6 Département Biomédical et Santé Publique, Institut de Recherche en Sciences de la Santé (IRSS/CNRST), Ouagadougou 03, 03 BP 7192 Burkina Faso

**Keywords:** Norovirus, Gastroenteritis, Meta-analysis, Epidemiology, Genotypes, Africa

## Abstract

Noroviruses are the second leading cause of death in children under the age of 5 years old. They are responsible for 200 million cases of diarrhoea and 50,000 deaths in children through the word, mainly in low-income countries. The objective of this review was to assess how the prevalence and genetic diversity of noroviruses have been affected by the introduction of rotavirus vaccines in Africa. PubMed, Web of Science and Science Direct databases were searched for articles. All included studies were conducted in Africa in children aged 0 to 5 years old with gastroenteritis. STATA version 16.0 software was used to perform the meta-analysis. The method of Dersimonian and Laird, based on the random effects model, was used for the statistical analyses in order to estimate the pooled prevalence’s at a 95% confidence interval (CI). Heterogeneity was assessed by Cochran’s Q test using the I2 index. The funnel plot was used to assess study publication bias. A total of 521 studies were retrieved from the databases, and 19 were included in the meta-analysis. The pooled norovirus prevalence’s for pre- and post-vaccination rotavirus studies were 15% (95 CI, 15–18) and 13% (95 CI, 09–17) respectively. GII was the predominant genogroup, with prevalence of 87.64% and 91.20% respectively for the pre- and post-vaccination studies. GII.4 was the most frequently detected genotype, with rates of 66.84% and 51.24% respectively for the pre- and post-vaccination studies. This meta-analysis indicates that rotavirus vaccination has not resulted in a decrease in norovirus infections in Africa.

**Supplementary Information:**

The online version contains supplementary material available at 10.1186/s12879-024-09434-6.

## Introduction

Viral gastroenteritis is the main cause of morbidity and mortality worldwide, with norovirus and rotavirus being the predominant viral agents responsible for diarrheal disease globally [1]. Norovirus is a significant global health concern, causing an estimated 200 million cases of diarrhoea and 50,000 deaths in children under 5 worldwide, with low-income countries experiencing the most severe impact. In children, noroviruses contribute to 14% of gastroenteritis cases globally, with 17% and 12% of hospital admissions occurring in low- and middle-income countries, respectively [2, 3]. The geographical distribution of norovirus remains variable, with a prevalence of 15% in Latin America [2], 17% in Asia (China) [4], and a global estimate of 19% in Africa among children under 5 years old [5].

Norovirus, discovered in 1968 [6], belongs to the Caliciviridae family and is a small, non-enveloped, single-stranded RNA virus [7, 8]. Its genome of approximately 7.6 kilobase pairs comprises three open reading frames (ORF). ORF1 encodes six non-structural proteins, while ORF2 and ORF3 code for the main and minor capsid proteins, respectively [9–11]. The VP1 component consists of S and P domains, with the P domain further divided into P1 and P2 sub-domains [12]. The VP2 protein, along with VP1’s S domain, aids in the virus’s capsid stability and assembly, potentially facilitating host cell attachment [9, 11, 13, 14].

Noroviruses are classified into genogroups, genotypes and variants (strains or sub-genotypes) [15]. There are 8 P-groups (GI.P to GVII.P and GX.P) and 60 P-types based on the nucleotide diversity of the RdRp region of ORF1 [16]. They are also classified into 10 genogroups (GI-GX) and 49 genotypes based on the VP1 capsid. Human-infecting genogroups include GI, GII, GIV, GVIII, and GIX [16], with GI and GII being the most prevalent [17].

These viruses enter the body targeting intestinal enterocytes, causing replication [18] and increased production of TNF-α and IL-6, leading to intestinal mucosa alterations [19], and clinical symptoms like diarrhoea, vomiting, abdominal pain, fever, and dehydration [19–22]. Humans are the only reservoir of human noroviruses, primarily transmitted through the faecal-oral route and potentially via contaminated aerosols [23]. Currently, there are no licensed vaccines or antiviral drugs for norovirus prevention [18]. The World Health Organization (WHO) prioritized norovirus vaccine development in 2016, with candidate vaccines based on non-replicable viral pseudoparticles (VLPs) in development [24]. Prevention efforts focus on hygiene and compliance measures, while treatment involves symptom management due to the challenges in vaccine development posed by the virus’s rapid genetic evolution, limited understanding of its pathogenesis, and transient protective immunity against enteric pathogens [8, 18, 25] [26].

Norovirus is responsible for sporadic cases and outbreaks of acute gastroenteritis in both children and adults, while rotavirus mainly affects neonates and children under 5 years of age. The WHO has recommended rotavirus vaccines in national immunization programs for children under 5 years old worldwide since 2007 [27, 28]. To date, four WHO-licensed vaccines (Rotarix, RotaTeq, Rotavac, and RotaSiil) effectively prevent rotavirus infections, notably reducing diarrhoea cases in children under 5 [29, 30]. The introduction of rotavirus vaccination is estimated to have reduced hospitalizations by 40% and annual deaths caused by rotavirus by 25%. However, anti-rotavirus vaccinations have shown lower effectiveness in developing countries, where acute gastroenteritis (AG) often leads to dehydration and malnutrition. Moreover, the widespread use of these vaccines has led to new challenges in combating gastroenteritis. Multiple studies across different countries have identified norovirus as the emerging primary cause of severe gastroenteritis in children following the global implementation of rotavirus vaccines. However, there is a scarcity of data and limited studies that have investigated this phenomenon in Africa [22, 31–33]. In this systematic review and meta-analysis, we focus on the evolution of norovirus prevalence and its genetic diversity before and after the implementation of rotavirus vaccines in Africa.

## Methodology

### Study search strategy

A literature search was conducted on electronic databases PubMed, Web of Science, and Science Direct using the following search terms: “Norovirus, Calicivirus, Norwalk virus, genotype, prevalence, epidemiology, rotavirus vaccines, gastroenteritis, children, diarrhoea, paediatric, and Africa” with no filters, language, or date restrictions. Boolean operators (AND, OR, NOT) were used to broaden or narrow searches during the literature review process. Two independent reviewers, DD and DO, screened titles and abstracts for relevance. The full-text articles were then obtained and assessed independently by these two reviewers for eligibility. Discrepancies were resolved by consensus, and if consensus couldn’t be reached, a third reviewer (AKO) was consulted. The reference lists of articles identified in the search were used to uncover additional literature.

### Inclusion and exclusion criteria for studies

We applied the following eligibility criteria: (1) studies conducted in African countries, (2) involving children aged 0 to 5 years, (3) with a minimum of 20 samples, 5) stool samples, 6) both before and after the introduction of anti-rotavirus vaccines, and 7) with no restrictions on publication dates. The period of anti-rotavirus vaccine implementation in each country was considered to determine pre- and post-vaccination studies. Information regarding the type of rotavirus vaccine, vaccination coverage, and vaccine introduction dates per country was obtained from the WHO through the International Vaccine Access Center [34]. Studies based on data from gastroenteritis surveillance systems and cross-sectional studies were included. Eligibility for inclusion in the present review was extended to any research that utilized polymerase chain reaction (PCR) and/or sequencing techniques, regardless of publication language (English or French).

Systematic reviews, conference abstracts, meta-analyses, editorials, clinical trial studies, letters, case reports, in vitro experiments and all studies not meeting the above selection criteria were excluded. Studies involving non-human subjects were excluded. Serological studies and those with inadequate data sets were excluded from the review. Inadequate data referred to studies that did not specify essential information such as the number of samples in different sub-populations (e.g., children in hospital, outpatients, community, ambulatory, with severe diarrhoea, without diarrhoea), the number of genotyped samples, or the number of successfully genotyped samples, etc.

### Study selection

**Fig. 1:**
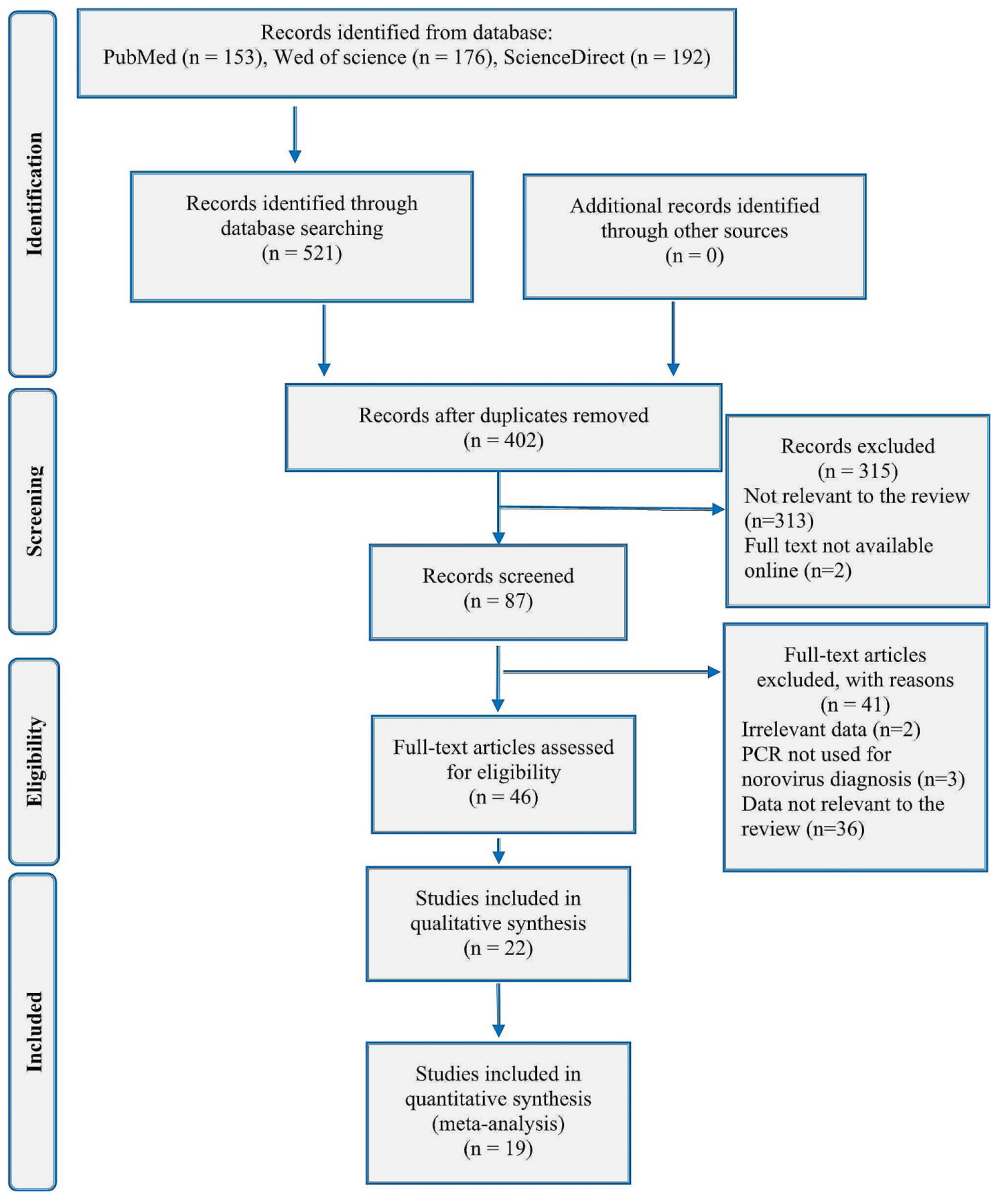
Flow chart for the selection of studies according to the PRISMA protocol

Upon completion of the search, the complete results from all databases were imported into a unique EndNote (version 20.2) library, and duplicates were subsequently removed. Reviewers initially screened studies by title and abstract in the EndNote library based on the inclusion criteria. Subsequently, the full text of relevant articles was retrieved if available online and subjected to the same screening process. A Preferred Reporting Items for Systematic Reviews and Meta-Analyses (PRISMA)-based study selection flowchart is shown in Fig. 1 [35].

### Data extraction

The relevant data from the selected studies were extracted by the reviewer DD and tabulated in a Microsoft Excel 2019 spreadsheet specifically developed for this review. The following data were extracted from each article when available: the name of the first author, the year of publication, the duration of the study, the country, the size of the study population, the diagnostic methods, the prevalence of norovirus in the study population and that of the different genogroups and genotypes, the number of positive cases, the size of the genotyped samples, the year of introduction of the rotavirus vaccine in the country where the study was conducted, the design of the study (cross-sectional study, sentinel surveillance study, etc.).

### Quality assessment: determining publication bias

To explore potential publication bias and test the assumption of symmetry among the included studies, the symmetry of the funnel plot was constructed. Egger’s objectivity estimation test was used with a *p* < 0.05 as evidence for the existence or not of publication bias [36].

### Data processing and analysis

Data from extracted from selected studies were entered into Microsoft Excel 2019 and imported into Stata software version 16.0 (Stata Corp., College Station, TX, USA) for statistical analysis [37, 38]. Microsoft Excel was used to construct frequency histograms illustrating the distribution of norovirus genotypes in children aged 0 to 5 years old in Africa. The following formulae were used to determine the prevalence:

- Prevalence of norovirus-positive cases in the study population = number of positive cases in/total number of samples tested.
- Genogroup prevalence = total number of positive cases of the genogroup concerned/total number of genogroups detected.
- Genotype prevalence = number of positive cases of the genotype concerned/total number of samples tested positive in the study or total number of genotyped samples.

The *metaprop* command in Stata software (Stata Corp., College Station, TX, USA) version 16.0 was used to perform the meta-analysis [37, 38]. DerSimonian and Laird’s random effects meta-analysis model [39] was used to generate pooled (combined) overall and subgroup prevalence estimates at 95% confidence intervals. These pooled prevalence’s are represented by forest plot graphs with 95% confidence intervals. Cochran’s Q test was used to determine the statistical heterogeneity index -I^**2**^ [37]. I^**2**^ values of 25%, 50%, and 75%, were considered low, moderate, and high levels of heterogeneity, respectively. Subgroup statistical analysis was performed for the pre- and post-vaccination studies and p-value < 0.05 was considered statistically significant [17].

## Results

### Literature search (analysis of primary data)

The search yielded 521 papers (PubMed: 167, Web of Sciences: 176 and Science Direct: 192). Studies identification included duplicates removing, title and abstract screening, and full-text screening leading to the selection of 19 articles for the systematic review and meta-analysis (Fig. 1). All the studies included in the meta-analysis were conducted between 2005 and 2022.

### Characteristics of the studies included in the systematic review and meta-analysis

Out of the 19 studies in the meta-analysis, 6 were from sentinel surveillance programs and networks for rotavirus and/or norovirus. Two of the three studies excluded from the meta-analysis did not perform genotypic characterisation of noroviruses [40, 41], and one could not be classified as a pre- or post-vaccination study [31]. The meta-analysis covered 14 African countries, with 6 (31.58%) studies from East Africa, 6 (31.58%) from West Africa, 3 (15.80) from Central Africa, 2 (10.52) from Southern Africa and 2 from North Africa. Tables 1 and 2 provide a summary of the characteristics of the studies included in the systematic review and meta-analysis.

**Table 1:** Characteristics of the studies included in the systematic review and meta-analysis

Reference	Country	Study period	Detection and genotyping method	Type of study	Type of population	Age of patients	Total population	Positive cases
Trainor et *al* [42].	Malawi	July 1997_June 2007	PCR in time	Cross-sectional	AGE/D hospitalised	**<** 5 years	1941	220
Rossouw et *al* [43].	South Africa	July 2016_December 2017	Multi-plex RT-PCR	Cross-sectional	Hospitalised for diarrhoea	**<** 5 years	205	32
Ouédraogo et *al* [44].	Burkina Faso	November 2011_September 2012	End-point RT-PCR	Cross-sectional	Consultants for AGE/Diarrhoea	**<** 5 years	263	55
Mugyia et *al* [21].	Cameroon	January 2010_December 2013	Conventional RT-PCR	Cross-sectional (Rotavirus sentinel surveillance programme).	AEDs hospitalised	**<** 5 years	902	76
Moyo et *al.* [45]	Tanzania	August 2010_July 2011	RT-PCR **+** conventional PCR	Foresight	Hospitalised for AGE/Diarrhoea	**<** 5 years	705	129
Makhaola et *al* [46].	Botswana	July 2013December 2015	Real-time multiplex PCR **+** conventional RT-PCR	Cross-sectional (Sentinel surveillance program for the impact of rotavirus vaccines).	Hospitalised for AEG/Diarrhoea	**<** 5 years	484	45
Kebe et *al.* [47]	Senegal	2007_December 2010	Real-time PCR **+** conventional RT-PCR	Cross-sectional	Consultants for AGE/Diarrhoea	**<** 5 years	599	79
Japhet et *al* [48].	Nigeria	August 2012_December 2013	RT-nested PCR	Cross-sectional	Hospitalised for AEG/Diarrhoea	0–5 years	103	11
Hungerford et *al.* [27]	Malawi	November 2012_December 2015	Single-plex real-time PCR	Cross-sectional (Rotavirus sentinel surveillance platform).	Consultants for AGE/Diarrhoea	**<** 5 years	684	42
Gelaw et *al* [49].	Ethiopia	November 2015_April 2016	Real-time RT-PCR	Cross-sectional (Rotavirus sentinel surveillance programme).	Hospitalised for Diarrhoea	**<** 5 years	450	60
El Qazoui et *al* [50].	Morocco	January_ December 2011	Real-time RT-PCR + conventional RT-PCR	Prospective (Regional rotavirus gastroenteritis surveillance network).	Hospitalised for AEG	**<** 5 years	335	57
Abugalia et *al.* [51]	Libya	October 2007_September 2008	Real-time RT-PCR	Cross-sectional	Inpatients + outpatients with AGE/Diarrhoea	**<** 5 years	520	91
Bonkoungou et *al* [52].	Burkina Faso	December 2012_November 2013	Modified TaqMan duplex real-time PCR	Cross-sectional (Active surveillance system for GEA/Severe diarrhoea)	Hospitalised for AGE	**<** 5 years	128	30
Dove et *al.*, 2005	Malawi	July 1998_June 1999	Conventional PCR	Cross-sectional	Hospitalised for AGE and dehydrated	**<** 5 years	398	26
Howard et *al* [53].	Zambia	July 2012_October 2013	RT-PCR	Cross-sectional (Rotavirus sentinel surveillance system)	Hospitalised for AGE/severe diarrhoea	0–59 months	454	52
Nordgren et *al.* [54]	Burkina Faso	May 2009_March 2010	Real-time PCR	Cross-sectional	People with AGE/Diarrhoea	**<** 5 years	309	37
Elfving et *al.* [40]	Zanzibar (Tanzania)	April-July 2011	Real-time PCR	Cross-sectional	People suffering from AGE/Diarrhoea and severe fever	2–59 months	156	34
Kabue et *al.* [41]	South Africa	July 2014_April 2015	Real-time RT-PCR	Cross-sectional	Ambulatory with diarrhoea	**<** 5 years	253	104
Rönnelid et *al.* [22]	Burkina Faso	January-December 2015	Real-time duplex PCR TaqMan	Cross-sectional (Rotavirus sentinel surveillance system)	Hospitalised for severe AGE	**<** 5 years	146	29
Esteves et *al* [55].	Angola	June 2012_October 2013	Internal real-time PCR **+** Multi-plex TaqMan real-time PCR	Cross-sectional	Hospitalised for acute diarrhoea	**<** 5 years	334	58
Mikounou Louya et *al* [56].	Democratic Republic of Congo	June 2012_June 2013	Nested RT-PCR **+** nested duplex RT-PCR	Cross-sectional (AGE surveillance system)	Hospitalised for acute diarrhoea	**<** 5 years	545	148
Lartey et *al.* [31]	Ghana	January 2008_December 2017	One Step RT-PCR	Cross-sectional (National rotavirus surveillance system)	Consultants for diarrhoea	**<** 5 years	1337	485

*Legend* Definition of abbreviations used in the table

AGE: Acute gastroenteritis

AGE/D: Acute gastroenteritis/diarrhoea

PCR: Polymerase Chain Reaction

RT-PCR: Reverse Transcription Polymerase Chain Reaction

RV1: Rotavirus vaccine (Rotarix), contains one strain of live attenuated human rotavirus (type G1P [8])

RV5: Rotavirus vaccine (RotaTeq), contains five reassortant strains developed from human and bovine parent rotavirus strains (G1P [5], G2P [5], G3P [5], G4P [5] et G6P [8])

**Table 2:** Characteristics of the studies included in the meta-analysis

Reference	Country	Sample collection period	Date of introduction of the rotavirus vaccine	Pre/post-vaccination study	Current rotavirus vaccine in use in the country	Vaccination coverage rate at the date of the study (%)	Coverage rate in 2023 (%)
Trainor et *al* [42].	Malawi	July 1997_June 2007	29 October 2012	Pre-vaccination	Rotarix (RV1)	-	92
Rossouw et *al.* [43]	South Africa	July 2016_December 2017	1 August 2009	Post-vaccination	Rotarix (RV1)	63	85
Ouédraogo et *al* [44].	Burkina Faso	November 2011_September 2012	31 October 2013	Pre-vaccination	RotaSiil (RV5)	-	91
Mugyia et *al* [21].	Cameroon	January 2010_December 2013	28 March 2014	Pre-vaccination	Rotarix (RV1)	-	65
Moyo et *al.* [45]	Tanzania	August 2010_July 2011	6 December 2012	Pre-vaccination	Rotarix (RV1)	-	77
Makhaola et *al* [46].	Botswana	June 2013_December 2015	3 July 2012	Post-vaccination	Rotarix (RV1)	85–92	85
Kebe et *al* [47].	Senegal	2007_December 2010	28 November 2014	Pre-vaccination	Rotarix (RV1)	-	84
Japhet et *al* [48].	Nigeria	August 2012_December 2013	22 August 2022	Pre-vaccination	Rotarix (RV1)	-	-
Hungerford et *al.* [27]	Malawi	November 2012_December 2015	29 October 2012	Post-vaccination	Rotarix (RV1)	7–84	92
Gelaw et *al.* [49]	Ethiopia	November 2015_April 2016	7 November 2013	Post-vaccination	Rotarix (RV1)	83–87	65
El Qazoui et *al* [50].	Morocco	January 2011_December 2011	20 October 2010	Post-vaccination	RotaTeq (RV5)	80	98
Abugalia et *al.* [51]	Libya	October 2007_September 2008	1^er^ October 2013	Pre-vaccination	RotaTeq (RV5)	-	73
Bonkoungou et *al* [52].	Burkina Faso	December 2012_November 2013	31 October 2013	Pre-vaccination	RotaSiil (RV5)	-	91
Dove et *al.* [57]	Malawi	July 1998_June 1999	29 October 2012	Pre-vaccination	Rotarix (RV1)	-	92
Howard et *al.* [53]	Zambia	July 2012_October 2013	26 November 2013	Pre-vaccination	Rotarix (RV1)	-	87
Nordgren et *al.* [54]	Burkina Faso	May 2009_March 2010	31 October 2013	Pre-vaccination	RotaSiil (RV5)	-	91
Rönnelid et *al.* [22]	Burkina Faso	January 2015_December 2015	31 October 2013	Post-vaccination	RotaSiil (RV5)	91	91
Esteves et *al.* [55]	Angola	June 2012_October 2013	28 April 2014	Pre-vaccination	Rotarix (RV1)	-	36
Mikounou Louya et *al* [56].	Democratic Republic of Congo	June 2012_June 2013	1 October 2019	Pre-vaccination	RotaSiil (RV5)	-	52

### Prevalence of norovirus

**Fig. 2:**
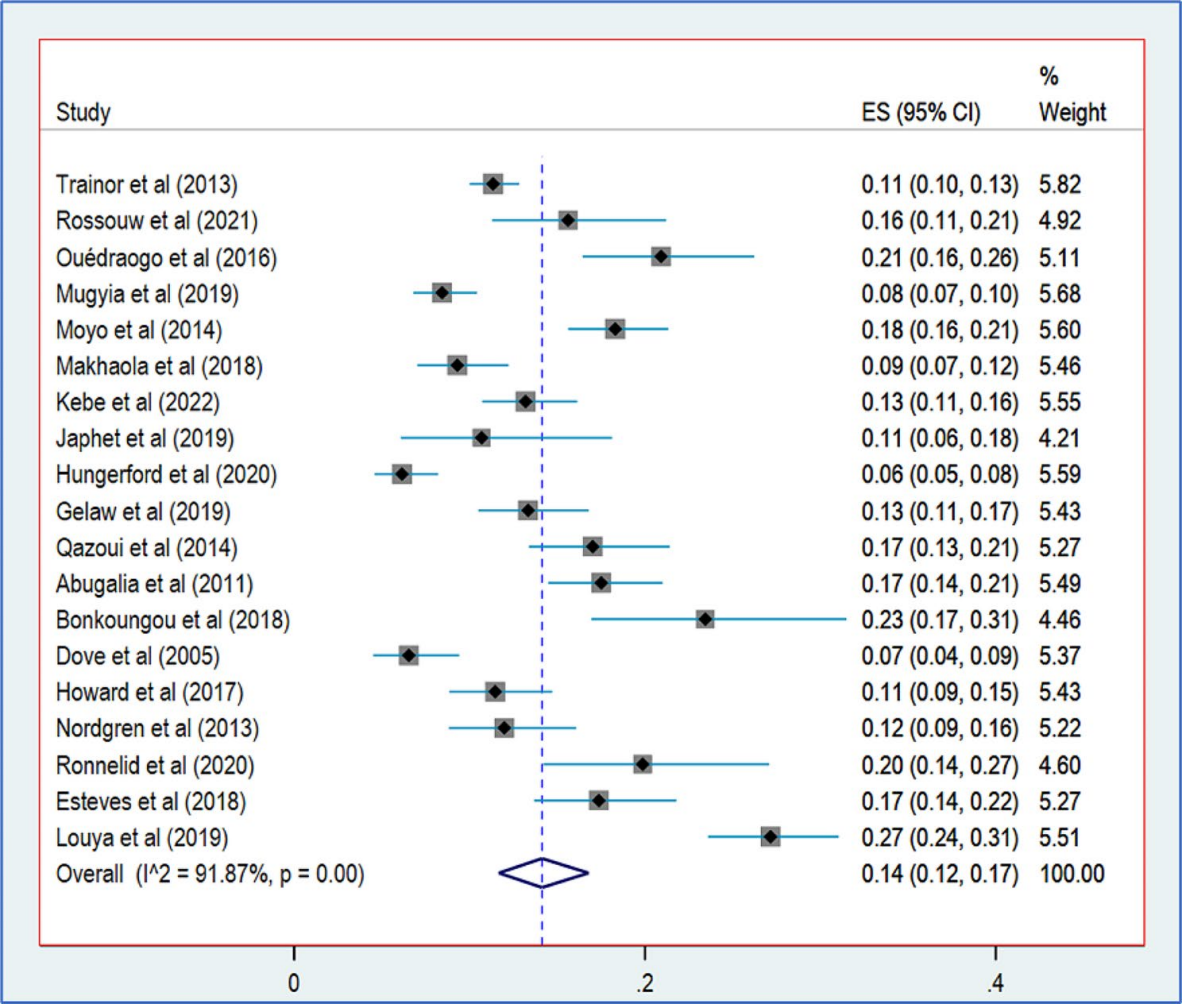
Forest plot of pooled norovirus prevalence in children aged 0–5 years with gastroenteritis in Africa. *Legend* The size of each grey square indicates the weight of the study contributing to the pooled prevalence estimate. The line running horizontally through each grey square refers to a 95% confidence interval with the mean effect at the centre (the point estimate of prevalence for each study). The dotted blue line represents the mean estimate of the pooled prevalence of norovirus. The dark blue diamond represents the 95% confidence interval of the pooled norovirus prevalence estimate. The length of the confidence interval is proportional to the size of the study sample and therefore to the precision of the estimate

Among the 19 articles in the meta-analysis, 9,505 samples from children aged 0–5 years with gastroenteritis were analysed. The cumulative prevalence of norovirus in children across all pre- and post-vaccination studies was 14% (95% CI, 12–17), with the forest plot (Fig. 2) indicating statistically significant heterogeneity (I^2^ = 91.87%, *p* < 0.001).

### Distribution of norovirus-positive cases by zone in Africa

**Fig. 3:**
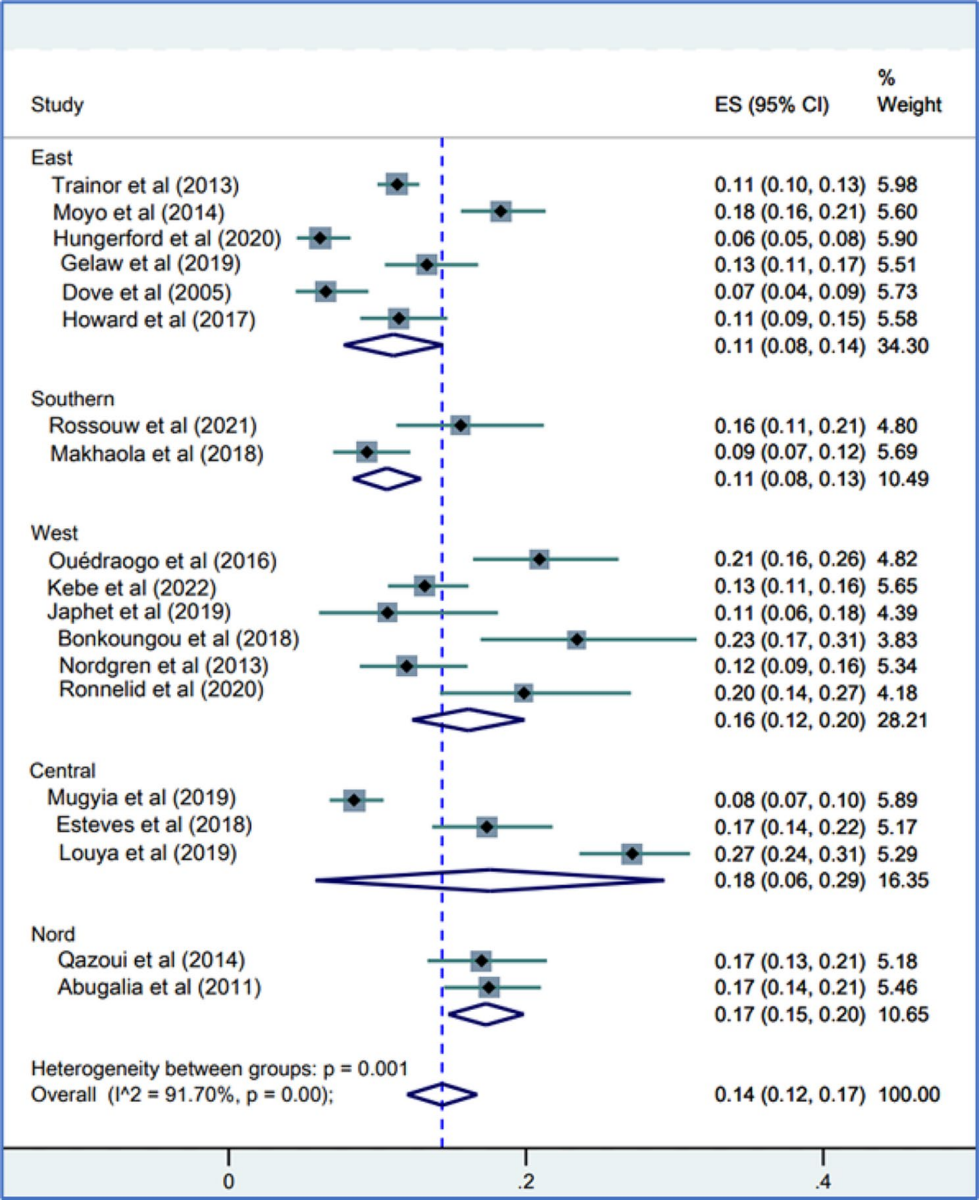
Distribution of norovirus infections in children aged 0–5 years by region of Africa

According to the distribution of norovirus infections by African zone, the highest prevalence was observed in Central Africa, with a value of 18% (95% CI, 6–29). North Africa had a prevalence of 17% (95% CI, 15–20). West Africa, with a prevalence of 16% (95 CI, 12–20). East Africa, with 11% (95 CI, 8–14) and South Africa, 11% (95 CI, 8–13), recorded the lowest prevalence of norovirus infections (Fig. 3).

### Subgroup statistical analyses

#### Norovirus prevalence in hospitalised and non-hospitalised children in Africa

Of the 19 studies included in this meta-analysis, 14 recruited only hospitalised children and 4 worked on samples from non-hospitalised children. One study that recruited both hospitalised and non-hospitalised children could not be classified because it did not specify the number of norovirus-positive cases in these two respective subgroups.

For the 14 studies that recruited hospitalised patients, a total of 7130 samples were tested compared with 1855 for non-hospitalised cases. The prevalence’s in these two subgroups of hospitalised and non-hospitalised children were 13.64% (973/7130) and 11.48% (213/1855) respectively.

#### Prevalence of norovirus before and after the introduction of rotavirus vaccines

Six studies included in this meta-analysis were post-vaccination studies against 13 pre-vaccination studies. The pooled prevalence of norovirus in children with gastroenteritis for pre-vaccination studies was 15% (95% CI, 15–18), and for post-vaccination studies, it was 13% (95% CI, 09–17). There was significant and important heterogeneity (Fig. 3) between the pre- (*p* < 0.001; I^2^ = 92.29%) and post-vaccination studies (*p* < 001; I^2^ = 89.53%).

**Fig. 4:**
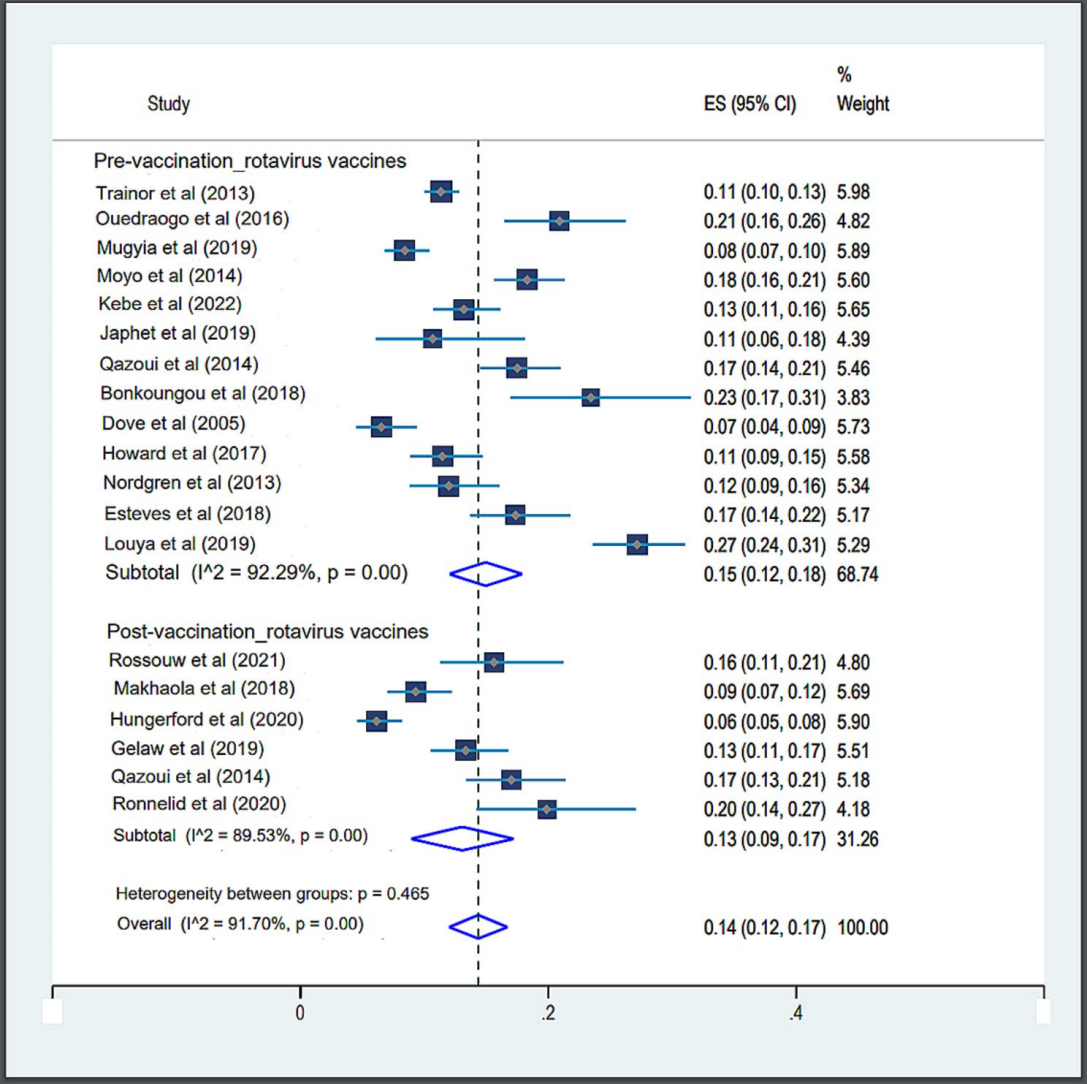
Forest representation of pooled prevalence estimates of norovirus infection before and after the introduction of rotavirus vaccines in Africa

### Diversity of norovirus genogroups in Africa

In the 19 studies included into this meta-analysis, 1277 samples tested positive for norovirus with successful genotyping of 1205. The overall prevalence of GII was 11.20% (135/1205), making it the predominant genogroup with a prevalence of 88.38% (1065/1205). GI.GII co-infection was observed at a prevalence of 0.33% (4/1205), while the GIX genogroup had the lowest reported prevalence (0.08%, 1/1205) in children with gastroenteritis. The cumulative prevalence of the genogroups during pre- and post-vaccination periods are provided in Table 3.

**Table 3:** Prevalence of norovirus genogroups in children aged 0–5 years with gastroenteritis in Africa

Genogroup	Number of studies	Pool prevalence
Pre- and post-vaccination studies	19	-
GI	18	11.20 (135/1205)
GII	19	88.38 (1065/1205)
GI.GII	1	0.33 (4/1205)
GIX	1	0.08 (1/1205)
Pre-vaccination studies against rotavirus	13	-
GI	10	11.94 (114/955)
GII	13	87.64 (837/955)
GI.GII	4	0.42 (4/955)
Post-vaccination studies against rotavirus	6	-
GI	5	8.4 (21/250)
GII	6	91.2 (228/250)
GIX	1	0.4 (1/250)

### Rotavirus vaccine and norovirus genotypic diversity in Africa

In 13 pre-vaccination studies, 955 norovirus-positive samples were identified, with 677 genotyped and 570 successfully analysed. In 6 post-vaccination studies, out of 250 positive samples, 201 were genotyped. The GII.4 genotype was most prevalent in both groups, with 66.84% in pre-vaccination and 51.24% in post-vaccination studies. Other significant genotypes included GII.3 (4.40%), GII.6 (3.80%), GI.3 (3.51%), GII.16 (3.00%), GII.2 (2.63%), GII.7 (1.61%), GII.21 (1.41%), GI.1 (1.40%), GI.5 and GII.17 (1.23% each) in the pre-vaccination studies and GII.6 (8.95%), GII.12 and GII.3 (5.47% each), GII.17 (4.97%), GII.10 and GII.12 (3.99% each), GII.17 (4.97%), GII.7 (2.98%), GI.3 and GII.13 (2.48% each) in the post-vaccination studies (Fig. 4). The GI.1, GI.2, GI.4, GI.7, GI.11, GI.14, GII.1, GII.11 and GII.15 found pre-vaccination were absent post-vaccination.

**Fig. 5:**
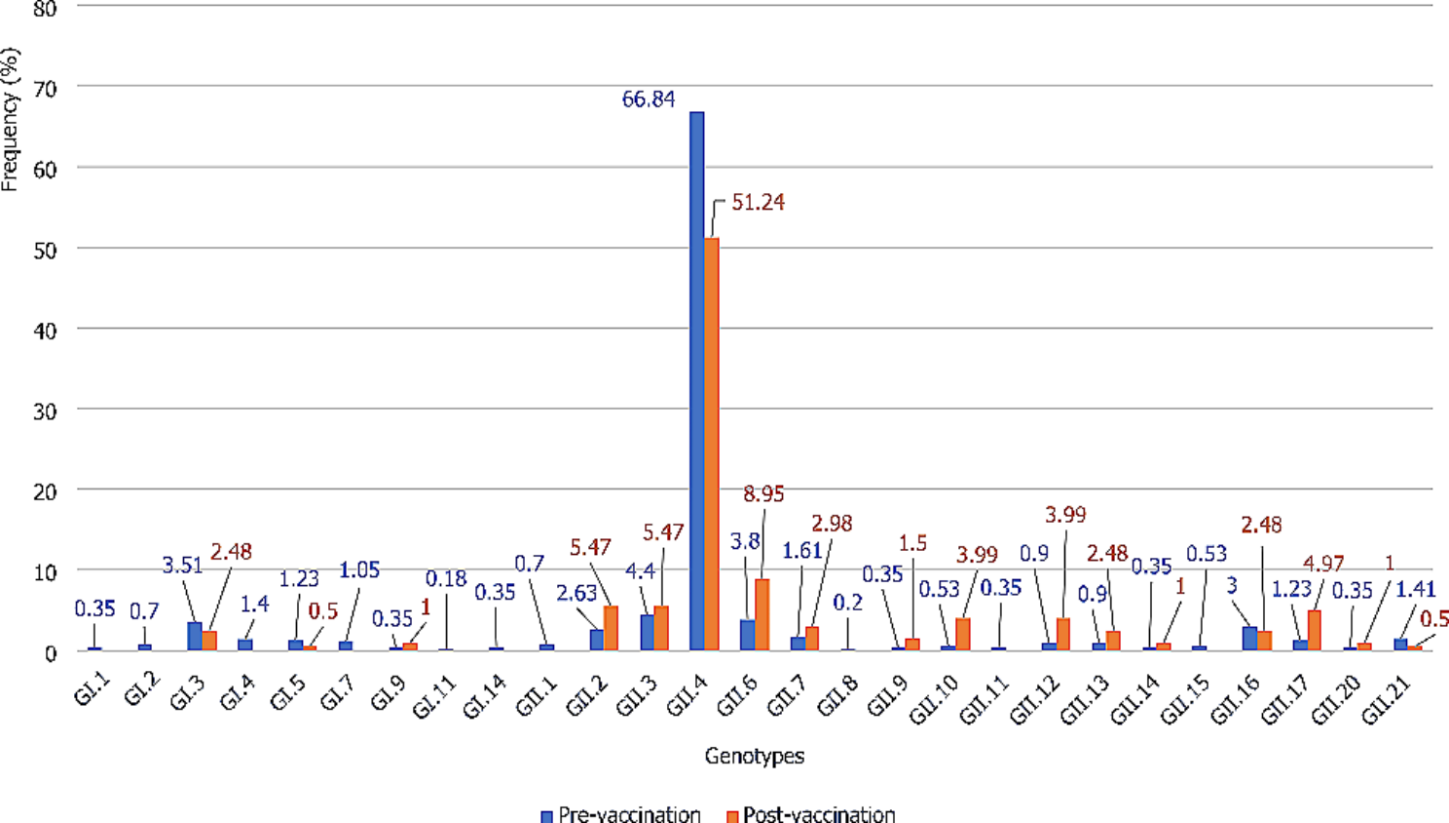
Distribution of norovirus genotypes in children aged 0–5 years with gastroenteritis in Africa

### Distribution of recombinant noroviruses

In the present study, recombinant norovirus infections were observed in four studies (Angola, Malawi, Senegal and Nigeria) [42, 47, 48, 57]. Recombinants reported included inter-genogroups (GI.3_GII.3 and GII.4_GI.5) and intra-genogroups (GII.4_GII.6 and GII.7_GII.14). The combined prevalence was 0.2% (1/570) for each of the four recombinants.

### Rotavirus and Norovirus co-infection in children

In this meta-analysis, only 8 studies including one post-vaccination study, assessed rotavirus infections in norovirus-infected children, with the combined prevalence of norovirus-rotavirus co-infection at 22% (95% CI, 15–29). The highest co-infection rate (36%, 95% CI, 15–65) was in Nigeria, the lowest (12%, 95% CI, 06–21) in Cameroon. Figure 5 illustrates a forest plot of these co-infection cases.

**Fig. 6:**
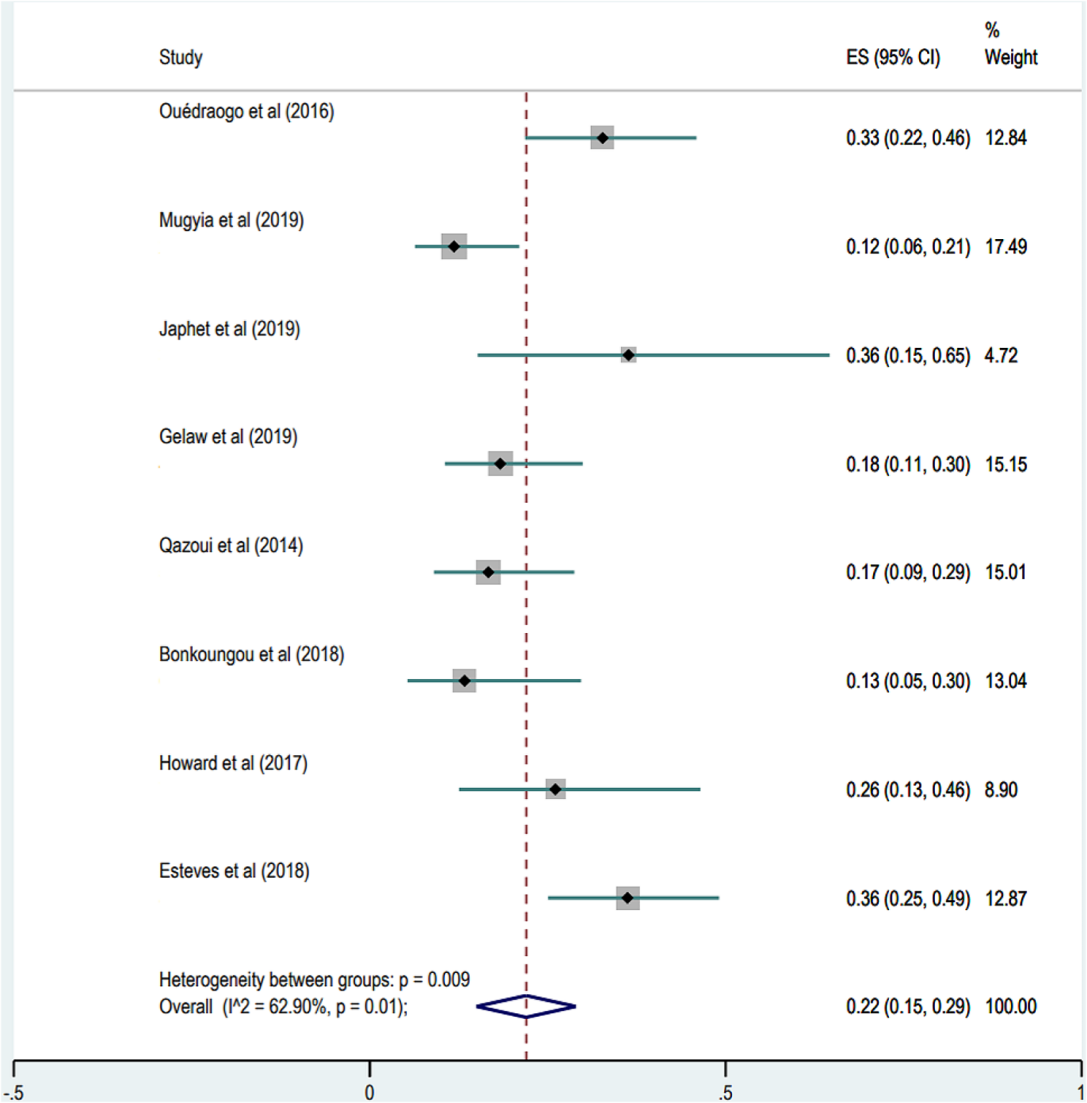
Forest plot of the distribution of cases of rotavirus-norovirus co-infections in African children aged 0–5 years

### Prevalence and genetic diversity of noroviruses in asymptomatic children in Africa

#### Prevalence of noroviruses in asymptomatic children in Africa

Of the 19 studies included in this meta-analysis, 5 recruited case controls. Controls were children aged between 0 and 5 years without gastroenteritis and without clinical manifestations, asymptomatic. Of the 5 studies that included controls, 3 were pre-vaccination studies and 2 were post-vaccination studies. For the 5 studies, the total number of children with gastroenteritis (symptomatic children) was 3,798 compared with 1,689 for asymptomatic children. The prevalence of norovirus (Table 4) in these two subpopulations of symptomatic and asymptomatic children was 12.5% (478/3798) and 8.70% (147/1689) respectively.

**Table 4:** Prevalence of noroviruses in asymptomatic children in Africa

	Symptomatic children			Asymptomatic children		
Reference	Population	Positive cases	Prevalence (%)	Population	Positive cases	Prevalence (%)
Trainor et *al.* (2013) [42],	1941	220	11.33	505	60	11.88
Rossouw et *al.* (2021) [43],	205	32	15.60	46	10	21.74
Ouédraogo et *al.* (2016) [44],	263	55	20.91	50	3	6.00
Moyo et *al.* (2014) [45],	705	129	18.30	561	52	9.27
Hungerford et *al.* (2020) [27],	684	42	6.14	527	22	4.17
Total population	3 798	1 689	-	478	147	-
Pool prevalence (%)	-	-	12.58	-	-	8.70

#### Genetic diversity of noroviruses in asymptomatic children in Africa

**Fig. 7:**
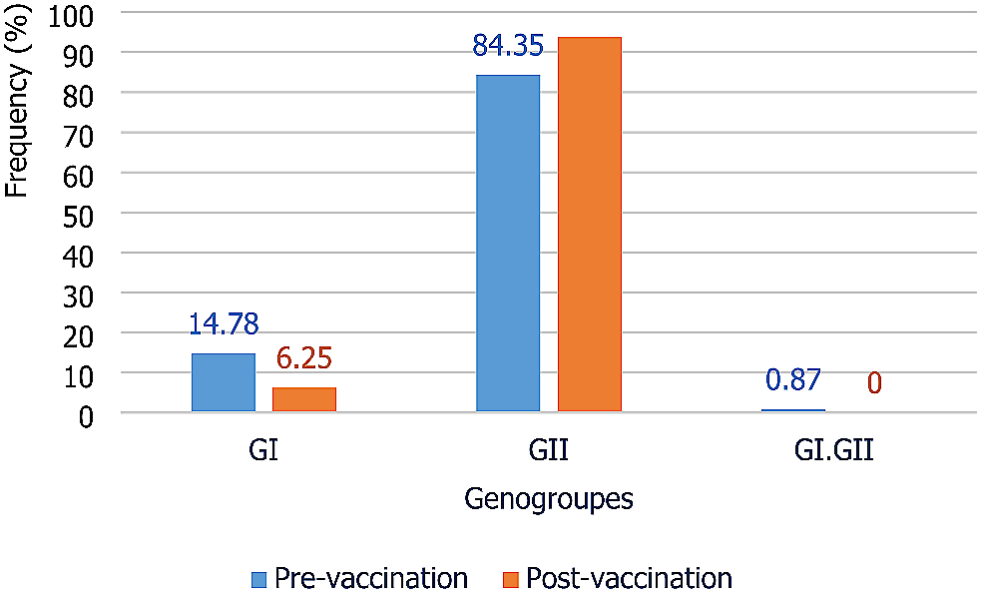
Prevalence of norovirus genogroups in children aged 0–5 years no gastroenteritis in Africa

Of the 147 norovirus positive samples, GI represented 12.92% (19/147) and recombinant GI.GII with a prevalence of 0.68% (1/147). GII was by far the most important genogroup, with a prevalence of 86.40% (127/147). Figure 7 shows the prevalence of the different norovirus genogroups in children aged 0–5 years without gastroenteritis. GII was the predominant genogroup in the pre- and post-vaccination studies, with prevalence’s of 84.35% (97/115) and 93.75 (30/32) respectively. GI was observed with proportions of 14.78% (17/115) and 6.25% (2/32) respectively in the pre- and post-vaccination studies.

#### For genotype detection, 59 were admitted for genotyping and 55 were successfully assigned

In the pre-vaccination studies, 5 (GI.5, GI.7, GII.4, GII.16 and GII.21) and 12 (GI.3, GI.7, GII.1, GII.2, GII.3, GII.4, GII.6, GII.10, GII.13, GII.14, GII.16 and GII.21) were observed. GII.4 was the most frequently detected genotype, with prevalence’s of 54.17% (13/24) and 38.71% (12/31) respectively for the pre- and post-vaccination studies.

**Fig. 8:**
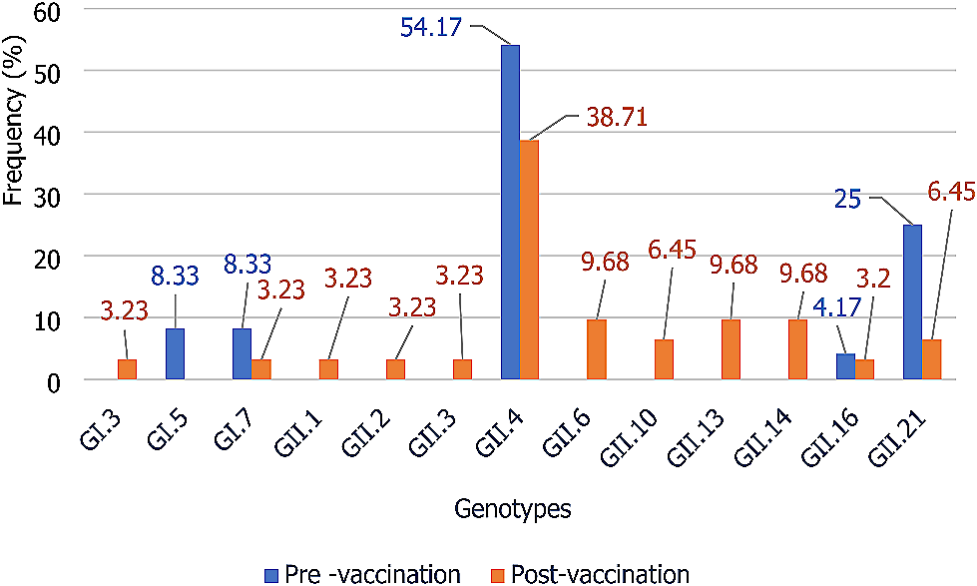
Distribution of norovirus genotypes in children aged 0–5 years no gastroenteritis in Africa

In the pre-vaccination studies, genotypes GII.21 (25%), GI.5 (8.33%) and GI.7 (8.33%) were the most frequently observed after GII.4. In the post-vaccination studies, GII.6, GII.13 and GII.14, with a prevalence of 9.68% each, were most frequently detected after GII.4 (Fig. 8).

### Evaluation of publication bias

**Fig. 9:**
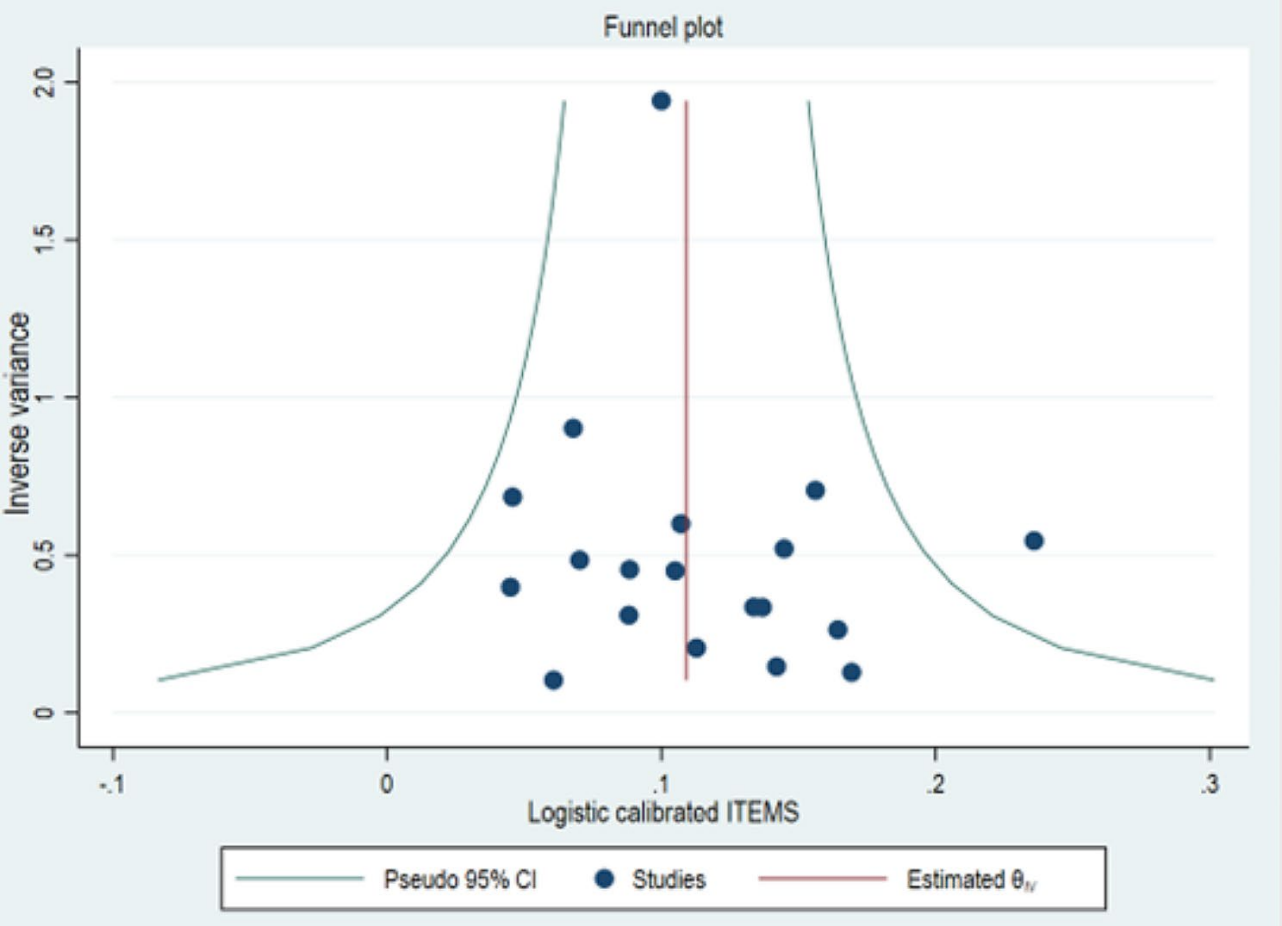
Funnel diagram for assessing study publication bias. ***Legend***: *Each point represents a study. The subjective evaluation of this diagram gives an indication of the heterogeneity of the studies. The y-axis represents the precision (inverse of the variance) expressed here at a ratio of 1/1000. The x axis represents the standardised effect transformed into a log (estimate divided by its standard error)*

The results of the graph showed a fairly low publication bias, as indicated by the distribution of studies in Fig. 9. Egger’s objectivity test confirmed the low level of publication bias in the studies selected for this meta-analysis.

## Discussion

Norovirus gastroenteritis is prevalent in low- and middle-income countries, with poor socio-economic conditions and sanitation [2]. In this meta-analysis, 9505 symptomatic gastroenteritis cases were examined across 19 studies. The pooled norovirus detection rate in children (0–5 years old) was 14% (95% CI, 12–17). These results align with global estimates of 14% (95% CI, 11–16) of norovirus infection in children with acute gastroenteritis [2, 3] and are close to the 15% reported in Latin America [58]. However, our findings contrast with Ahmed et *al.* [3] meta-analysis who found norovirus prevalence of 18% (95% CI, 15–20) in children under 5 years old. Other meta-analyses focusing on children under 5 in Africa and Asia (China) reported norovirus prevalence of 19.25% (95% CI, 14.4–23.5) [5], and 17.39% (95% CI, 17.32–17.47) [4], respectively. Additionally, a study in low- and middle-income countries found a 17% prevalence (95% CI, 17–18) of norovirus among hospitalised children under 5 years [2]. The discrepancies in norovirus prevalence between various studies could be attributed to differences in detection method sensitivity, some are more sensitive than others [59], geographical areas [49], and duration of study periods [5]. Notably, a higher burden of norovirus diarrhoea during the dry season has been observed [60]. In our meta-analysis, substantial heterogeneity was observed among the included studies, indicating significant variation. In addition to the sensitivity of the different detection methods used by the included studies, the geographical area and the length of the study periods, other reasons could explain this heterogeneity. These include the limited number of sampling sites found in the included studies, the short sampling period, and the small sample sizes for most of the included studies. The limited sample sizes make it impossible to capture broad information on the genetic diversity and national circulation of noroviruses. The context in which the studies were conducted, and the definition of cases of diarrhoea or gastroenteritis are other factors that could explain the heterogeneity observed between the studies included in this meta-analysis. Policies on the management of gastroenteritis differ from country to country. Most children suffering from gastroenteritis do not routinely undergo tests to establish aetiology, except in cases of spontaneous studies and severe gastroenteritis.

The pooled prevalence’s of norovirus for the pre- and post-vaccination studies were 15% (95% CI, 15–18) and 13% (95% CI, 13–17) respectively. For pre-vaccine studies, our observations were similar to those of O’Ryan et *al*. [61] who reported a pooled prevalence of 15% (95% CI: 12–19) for their rotavirus pre-vaccine studies. The pooled post-vaccination prevalence found in this meta-analysis tends towards the 16% (95% CI: 12–22) reported by O’Ryan et al. [61]. In this study, we observed a change in the prevalence of norovirus from 15% (95% CI, 15–18) in the pre-vaccination period to 13% (95% CI, 13–17) in the post-vaccination period. However, given the heterogeneity of the studies included, this does not allow us to conclude that there has been a real reduction in the prevalence of norovirus following the introduction of rotavirus vaccines in Africa.

However, some studies conducted in low- and middle-income countries have found an increase in the pattern of norovirus infections after the introduction of rotavirus vaccines [31, 33, 61]. The slight decrease in norovirus infections observed in our study could be explained by the positive effect of raising awareness of norovirus prevention and control through networks (such as NoroNet and CaliciNet) and paediatric viral gastroenteritis surveillance programmes set up in the various African countries. This meta-analysis shows that the widespread use of rotavirus vaccines does not appear to have a real positive impact on the norovirus infection pattern in Africa. For the pre-vaccination studies, the combined prevalence’s for GII and GI were 87.64% and 11.94% respectively. These observations are consistent with previous studies conducted in Asia, Europe, Latin America, and Africa, which reported prevalence ranging from 72.1 to 97.8% for GII, and from 3.02 to 27.90% for GI [53, 62–64]. In post-vaccination studies, the combined proportions of GII and GI genogroups were 91.20% and 8.40% respectively. These results are similar to those of previous studies conducted in children under 5 years in Africa [31, 65].

Norovirus GII is the predominant norovirus genogroup worldwide [65, 66]. Our findings align with this observation, suggesting that GII predominance may be associated with its higher viral load in patient stools compared to GI [63]. Such a high concentration of GII would therefore enhance contagiousness. The ability of GII to escape the immune system, its affinity for histological blood group antigens (HBGA) and its ability to bind to cell receptors [63], are other factors that could explain the predominance of this genotype.

In this study, a wide genotypic diversity of noroviruses was observed. A total of 9 GI genotypes (GI.1–5,7,9,11, and GI.14) and 18 GII genotypes (GII.1–4, GII.6–17, GII.20 and GII.21) were identified in the pre-vaccination studies. In the post-vaccination studies, 3 genotypes (GI.3,5,9) of GI and 14 genotypes (GII.2–4, GII.6,7,9,10, GII.12–14, GII.16,17,20,21) of GII were detected. The genotypic diversity observed in this analysis is consistent with the results of other studies carried out in Africa and other parts of the world [4, 5, 63, 67–69]. Genotypic diversity is a well-documented feature of norovirus epidemiology [68, 70]. Indeed, noroviruses, like all RNA viruses, are naturally diverse [31]. Other factors such as hygiene, sanitation, and socio-economic conditions, are thought to contribute to the genetic diversity of noroviruses [71]. The distribution of the different genotypes shows that GII.4 is by far the predominant genotype in both pre- and post-vaccination studies, with combined prevalence of 66.84% and 51.24% respectively. These results are similar to those of previous studies conducted in Africa, which reported GII.4 prevalence of 54.1% [68] and 65.2% [67]. Other recent meta-analysis reported similar prevalence of 59.3% [65] and 52% [66].

For post-vaccination studies, our results are comparable to those of a meta-analysis on studies published between 2015 and 2020, with combined prevalence of 50.81% for GII.4 [5]. However, the prevalence of GII.4 observed in our study is lower than that (89.2%) of a study conducted in Brazil [72]. In Africa and other parts of the world, GII.4 has been shown to be the predominant genotype of all norovirus genotypes [5, 65, 66]. Our observation supports the conclusion that GII.4 has dominated over all non-GII.4 genotypes over the last two decades [21, 73]. The predominance of GII.4 is attributed to its high mutational capacity [74], driven by specific recombination [75]. GII.4 can accumulate mutations, periodically replacing antigenic variants [70], fostering the emergence of new variants capable of evading immune responses developed against previous ones [76].

In the present meta-analysis, genotypes GII.3 (4.40%), GII.6 (3.80%), GI.3 (3.51%), GII.16 (3.00%), GII.2 (2.63%), GII.7 (1.61%), GII.21 (1.41%), GI.1 (1.40%), GI.5 and GII.17 (1.23% each), were the most important genotypes in the pre-vaccination studies after GII.4. In the post-vaccination studies, genotypes GII.6 (8.95%), GII.12 and GII.3 (5.47% each), GII.17 (4.97%), GII.10 and GII.12 (3.99% each), GII.17 (4.97%), GII.7 (2.98%), GI.3 and GII.13 (2.48% each) were the most frequently detected after GII.4. These observations are similar to those of previous studies conducted in Africa and other parts of the world [5, 31, 33, 72, 77]. Non-GII.4 norovirus genotypes have a limited number of variants that can persist for decades [70]. This would explain their low prevalence compared to GII.4. The distribution of genotypes shows an emergence of GII.17, GII.6, GI.10, GII.12, GII.2, GII.13, GII.7, GI.9, and GII.3 genotypes after the introduction of rotavirus vaccines in Africa. This observation is similar to those of previous studies conducted worldwide, which have reported the emergence of GII.6, GII.17, GII.2, GII.3 [5], GII.2 [78–80], and GII.6 [81].

The emergence or re-emergence of genotypes GII.17, GII.6, GI.10, GII.12, GII.2, GII.13, GII.7, GI.9, and GII.3 and the predominance of GII.4, GI.3, GII.16, GII.7, GII.21, GI.1 and GI.5, observed in this study, shows the need to strengthen genomic surveillance of noroviruses in Africa. Genotypes GII.4, GI.3, GII.16, GII.7, GII.21, GI.1 and GI.5 could be the genotypes to target in the development of future norovirus vaccines.

In asymptomatic children, the pooled prevalence of norovirus infection was 8.70%. The pooled prevalence observed in this meta-analysis shows that asymptomatic children could play an important role in the transmission pattern of noroviruses in Africa. In this meta-analysis, genogroups GII and GI were observed at prevalence’s of 86.40% and 12.92% in asymptomatic children. As in symptomatic children, GII was by far the predominant genotype in asymptomatic children.

In the pre-vaccination studies, 5 (GI.5, GI.7, GII.4, GII.16 and GII.21) and 12 (GI.3, GI.7, GII.1, GII.2, GII.3, GII.4, GII.6, GII.10, GII.13, GII.14, GII.16 and GII.21) were observed. GII.4 was the most frequently detected genotype, with prevalence’s of 54.17% and 38.71% respectively for the pre- and post-vaccination studies. As with symptomatic children, a high genetic diversity of noroviruses was observed in asymptomatic children. In addition to the contexts in which the studies were carried out [5, 60], the geographical areas [49] and the different norovirus detection methods used by the different studies [59], the genetic diversity observed in this meta-analysis could also be explained by genetic variations linked to the mutation and recombination phenomena affecting the norovirus.

The results of this study will contribute to understanding the impact of rotavirus vaccines on norovirus epidemiology in Africa. This meta-analysis made it possible to estimate the evolution of the different norovirus genotypes circulating in Africa before and after the advent of rotavirus vaccines. This information could help to better guide norovirus prevention strategies, particularly with regard to the genotypes to target, in the development of future norovirus vaccines. The results of this meta-analysis could therefore help in the development of vaccines that will be more effective in the real socio-economic context of the African region. The results of this study also serve as a reminder of the need to step up sentinel surveillance against noroviruses in Africa.

### Limitations of the study

Heterogeneity between the studies included in this meta-analysis could have an effect on the overall grouped prevalence or those of the subgroups. This heterogeneity could be related to differences in the spatio-temporal and epidemiological patterns of the studies included in this meta-analysis. Also, although seasonality of norovirus infections is important for effective healthcare planning, in our study we were unable to establish seasonality because it was inconsistent across individual studies. This is another limitation of this study.

## Conclusion

This meta-analysis provided interesting, up-to-date data and information on the evolution of the prevalence, molecular epidemiology, and genetic diversity of noroviruses in children aged 0 to 5 years old after the introduction of universal rotavirus vaccination program in Africa. Our study confirmed the important role played by noroviruses in gastroenteritis in young children both before and after the introduction of rotavirus vaccines in Africa. The advent of rotavirus vaccines in Africa has not shown any real positive impact on norovirus infections. While waiting for norovirus vaccines to become available, it will be necessary to strengthen paediatric gastroenteritis control systems and strategies in Africa.

### Electronic supplementary material

Below is the link to the electronic supplementary material.

Supplementary Material 1

## Data Availability

The datasets used and/or analyzed during the current study are available from the corresponding author on reasonable request.
