# Supplementary material for: Rotavirus vaccines in Africa and Norovirus genetic diversity in children aged 0 to 5 years old: a systematic review and meta-analysis: Rotavirus vaccines in Africa and Norovirus genetic diversity
Source: BMC Infect Dis. 2024 May 31;24:547. doi: 10.1186/s12879-024-09434-6 (PMC11143598; doi:10.1186/s12879-024-09434-6)
Supplement: Supplementary file 1 — Supplementary Material 1 [file 12879_2024_9434_MOESM1_ESM.docx]

**Supplementary file**

The combinations of keywords used to search for studies in the electronic databases are shown below.

TI = (Norovirus* OR *Calicivirus* OR *Norwalk virus*)* AND (genotype* OR rotavirus vaccine* OR epidemiology OR gastroenteritis OR prevalence* OR pediatric* OR diarrhea* OR children) AND Africa))

AB = (Norovirus* OR *Calicivirus* OR *Norwalk virus*)* AND (genotype* OR rotavirus vaccine* OR epidemiology OR gastroenteritis OR prevalence* OR pediatric* OR diarrhea* OR children) AND Africa))

Norovirus[tiab] AND (genotype OR prevalence OR epidemiology OR rotavirus vaccine OR gastroenteritis, OR diarrhea OR children) AND "Africa"[Mesh]

*Calicivirus* [tiab] AND (genotype OR prevalence OR epidemiology OR rotavirus vaccine OR gastroenteritis, OR diarrhea OR children) AND "Africa"[Mesh]

*Norwalk virus* [tiab] AND (genotype OR prevalence OR epidemiology OR rotavirus vaccine OR gastroenteritis, OR diarrhea OR children) AND "Africa"[Mesh]

Norovirus[tiab] OR *Calicivirus*[tiab] OR Norwalk virus[tiab] AND (genotype OR prevalence OR epidemiology OR vaccine OR gastroenteritis OR children OR diarrhea OR pediatric) AND "Africa"[Mesh] »
